# Current Status of Outdoor Lifetime Testing of Organic Photovoltaics

**DOI:** 10.1002/advs.201800434

**Published:** 2018-06-10

**Authors:** Yiwei Zhang, Ifor D. W. Samuel, Tao Wang, David G. Lidzey

**Affiliations:** ^1^ Organic Semiconductor Centre SUPA School of Physics and Astronomy University of St Andrews St Andrews KY16 9SS UK; ^2^ School of Materials Science and Engineering Wuhan University of Technology Wuhan 430070 China; ^3^ Department of Physics and Astronomy University of Sheffield Sheffield S3 7RH UK

**Keywords:** lifetime, organic photovoltaics, outdoor, stability, test protocols

## Abstract

Performance degradation is one of the key obstacles limiting the commercial application of organic photovoltaic (OPV) devices. The assessment of OPV stability and lifetime are usually based on simulated degradation experiments conducted under indoor conditions, whereas photovoltaic devices experience different environmental conditions under outdoor operation. Besides the intrinsic degradation of OPV devices due to the evolution of optoelectronic and morphological structure during long‐term operation, outdoor environmental changes can impose extra stresses and accelerate the degradation of OPV modules. Although outdoor studies on long‐term OPV stability are restricted by the long data collection times, they provide direct information on OPV stability under mixed degradation stresses and are therefore invaluable from the point of view of both research and practical application. Here, an overview of the current status of outdoor lifetime studies of OPVs is provided. After a summary of device lifetime extrapolated from indoor studies, outdoor lifetime testing platforms are introduced and the operational lifetime of various OPV devices are reviewed. The influence of climate and weather parameters on device performance and burn‐in phenomena observed during the degradation of OPVs is then discussed. Finally, an outlook and directions for future research in this field are suggested.

## 1 Introduction

Organic photovoltaic (OPV) devices are a candidate for next generation photovoltaic (PV) applications because they can be solution‐processed on light‐weight, flexible substrates over large areas:^1^ a property that could greatly decrease manufacturing cost and permit new applications such as wearable devices. OPVs also have the potential for shorter energy payback times compared to many other PV technologies as a result of lower embodied energy in the solution‐based deposition techniques that are expected as part of their manufacture.^2^

The past decade has witnessed a rapid improvement in OPV efficiency. Through the combined effort of chemical design and synthesis, new polymer donors and nonfullerene organic semiconductor acceptor materials have emerged and enabled numerous photovoltaic blend systems to achieve power conversion efficiencies (PCE) in excess of 10%;^3^ a level considered as a milestone for commercialization. However, high efficiency is not the only requirement for commercialization; rather extended operational stability also must be demonstrated. For silicon based PVs (the technology that presently dominates the PV market), operational stabilities of 20 years can be achieved.^4^ For OPVs, it has been estimated that a lifetime of at least 10 years must be demonstrated to render such devices financially competitive; a level of stability that currently remains challenging.

The degradation of OPV device performance has been widely observed, however the volume of research undertaken to study this process is substantially less than that devoted to the development of new materials or processing studies undertaken to engineer an enhancement in PCE.^5^ Known degradation mechanisms include photo‐ and water‐induced chemical reactions within the active layer, the degradation of device electrodes, the instability of hole and electron transport layers and a failure of device encapsulation. A detailed discussion of device degradation mechanisms can be found in a number of comprehensive reviews.[[qv: 5b,6]]

Compared to outdoor studies, lifetime studies conducted under indoor conditions combine the advantages of reduced data collection time together with well‐controlled and well‐defined environmental conditions. However the degradation pathways that exist during indoor studies are usually fixed rather than evolving dynamically, and thus outdoor lifetimes cannot be easily predicted by linear extrapolation of different degradation factors. Rather, outdoor lifetime testing can directly provide information regarding OPV module stability under real world conditions that change dynamically.

In this review, we start with a brief introduction to indoor lifetime testing. Following this, long‐term outdoor lifetime setups are introduced, with a comparison of outdoor lifetime studies on a series of OPV devices presented. We focus on factors that have been found to affect OPV degradation, including temperature, irradiance level, humidity, and thermal cycling. Morphological and optoelectronic contributions to device burn‐in and recent reports of burn‐in free systems are also discussed.

## 2 OPV Lifetime Extrapolated from Indoor Lifetime Studies

Outdoor real‐world lifetime studies of OPVs are time consuming and require a comprehensive testing platform. Because of this, the lifetime of OPV devices is usually extrapolated from indoor degradation tests that are run under accelerated conditions.^7^

Before 2011, there were no specific standards for OPV lifetime testing, and thus the results reported before then cannot be fully compared due to differences in data collection, analysis and presentation methods. At that time, the standards used in some OPV lifetime research were based on protocols developed by the International Electrotechnical Committee (IEC) for the characterization of amorphous silicon PVs. Here the most commonly used standard is known as IEC61646 which comprises a series of degradation tests, including a 1000 h damp heat (DH) test at 85 °C and 85% humidity, 200 cycles of thermal cycling (TC) from −40 to +85 °C, and a sequence test consisting of UV exposure, 50 cycles of TC, and 10 cycles of humidity freeze (HF) from −40 to +85 °C at 85% humidity. After finishing each test, modules are then characterized to determine device efficiency.

The feasibility of applying the IEC61646 standard to OPV lifetime testing has been explored. For example, Yan et al.^8^ characterized the stability of semitransparent OPV modules based on P3HT:PCBM following the IEC61646 standard. They found that modules with an initial efficiency of around 3% underwent an efficiency loss of 8% for modules encapsulated using a flexible barrier and 4% for laminated glass encapsulation by the end of the test period. However as the IEC61646 standard was established for amorphous silicon thin film solar cells; there are concerns regarding its application to OPVs; as the degradation mechanisms active in silicon based photovoltaics and OPVs are unlikely to be the same. For this reason an International Summit on Organic solar cell Stability (ISOS) was held in 2011 and discussed issues relating to the reliability and repeatability of OPV lifetime studies. Following this, recommendations for OPV stability tests were established based on the consensus of a large number of research groups that now provide standards for the study of OPV stability, allowing a direct and more reliable comparison to be made between different research studies.^9^

OPV lifetime testing conducted under laboratory conditions can be divided into several conditions, with devices being subjected to dark storage, laboratory‐weathering, thermal‐cycling and solar‐thermal‐humidity cycling. For each test, three levels are defined according to the requirements for measurement facilities and accuracy, as shown in **Table** **1**.

**Table 1:** Summary of lifetime testing types and conditions. Adapted with permission.^9^ Copyright 2011, Elsevier

Level	Description					
Basic (1)	Manual measurements using simple equipments and few conditions					
Intermediate (2)	Fixed conditions and protocols suitable for most laboratories					
Advanced (3)	Standardized tests applied in certified laboratories. Extended range of parameters to be monitored					
Test type	Test ID	Light source	Temp.	Relative humidity	Environment	Light source
Dark	ISOS‐D‐1 shelf	None	Ambient	Ambient	Ambient	Solar simulator or sunlight
	ISOS‐D‐2 high temp. storage	None	65 °C/85 °C	Ambient (low)	Oven	Solar simulator
	ISOS‐D‐3 damp heat	None	65 °C/85 °C	85%	Environ. chamber	Solar simulator
Laboratory weathering testing	ISOS‐L‐1 laboratory weathering	Simulator	Ambient	Ambient	Light only	Solar simulator
	ISOS‐L‐2 laboratory weathering	Simulator	65 °C/85 °C	Ambient	Light and controlled temp.	Solar simulator
	ISOS‐L‐3 laboratory weathering	Simulator	65 °C/85 °C	Around 50%	Light and controlled temp. and R.H.	Solar simulator
Thermal cycling	ISOS‐ T‐1 thermal cycling	None	Between R.T. and 65 °C/85 °C	Ambient	Hot plate/oven	Solar simulator or sunlight
	ISOS‐ T‐2 thermal cycling	None	Between R.T. and 65 °C/85 °C	Ambient	Oven/environ. chamber	Solar simulator
	ISOS‐ T‐3 thermal cycling	None	Between −40 °C and +85 °C	Around 55%	Environment chamber	Solar simulator
Solar‐thermal‐humidity cycling	ISOS‐LT‐1 solar‐thermal cycling	Simulator	Linear or step ramping between R.T. and 65°C	Monitored and uncontrolled	Weather chamber	Solar simulator
	ISOS‐LT‐2 solar‐thermal‐humidity cycling	Simulator	Linear ramping between 0 and 65 °C	Monitored and controlled at 50% beyond 40 °C	Environment chamber with sun simulation	Solar simulator
	ISOS‐LT‐3 solar‐thermal‐humidity‐freeze cycling	Simulator	Linear ramping between −25 °C and +65°C	Monitored and controlled at 50% beyond 40 °C	Environment chamber with sun simulation and freezing	Solar simulator

Dark storage and laboratory weathering tests are two widely used long‐term lifetime tests that are conducted indoors. Thermal cycling and solar‐thermal‐humidity are rarely applied due to the relative complexity of the tests as well as the short lifetime of most OPVs under such harsh conditions.

In dark storage tests, OPV devices are simply stored in the dark, with the exposure to atmospheric oxygen and moisture being the main degradation processes. According to ISOS test protocols, devices can be exposed to ambient or elevated temperatures and humidity, with tests corresponding to ISOS‐D‐1, ISOS‐D‐2, and ISOS‐D‐3 tests as described in Table 1. Angmo and Krebs^10^ fabricated large area, ITO‐free P3HT:PCBM OPV devices using roll‐to‐roll techniques and investigated long‐term dark‐storage lifetime following the ISOS‐D‐2 standard. It was found that OPV modules retained more than 80% of their initial efficiency after more than 2 years dark‐storage, with the efficiency loss being mostly attributed to degradation at the electrode contacts. Although the initial efficiency of the above P3HT:PCBM modules was relatively low (PCE of 1.06%), such results are very encouraging considering that the modules were fabricated using scalable techniques and indicate a promising stability of the organic photo‐active layer against atmospheric oxygen at elevated temperatures and low humidity. Fullerene and nonfullerene based OPVs with higher initial efficiencies have also been tested employing ISOS‐D standards (see **Table** **2**). In recent years, following the rapid development of perovskite solar cells (PSCs), ISOS‐D standards have also been applied to investigate the stability of such devices.^11^ Generally, dark storage lifetime studies are employed to determine the stability of OPV devices when exposed to air with or without extra thermal or moisture stresses. Since photo‐induced chemical reactions do not occur during dark storage, degradation under this type of test is usually attributed to the ingress of oxygen and water into the device; a process that often results in the failure of the device contact or degradation of the photoactive layer. Such degradation mechanisms also occur under outdoor conditions and thus indoor testing provides important information regarding device stability, despite its inability to provide a precise measure of OPV stability under real‐world conditions.

**Table 2:** Lifetime of various OPVs obtained using different indoor testing protocols

Dark storage lifetime					Constant irradiance lifetime				
Test protocols	Active layer	Initial PCE [%]	Lifetime	Ref.	Test protocols	Active layer	Initial PCE [%]	Lifetime [h]	Ref.
ISOS‐D‐1	P3HT: PCBM	3.14	245 days	16	ISOS‐L‐1	DR3TSBDT: PC_70_BM	9.6	5600	17
ISOS‐D‐3	P3HT: PCBM	2.7	>12 000 h	18	ISOS‐L‐1	PCDTBT: PC_70_BM	5.5	>12 000	14
ISOS‐D‐1	PTB7: PC_70_BM	7.76	<300 h	19	ISOS‐L‐1	PCDTBT: PC_70_BM	5.5	≈18 000	20
ISOS‐D‐2	P3HT: PCBM	≈1.6	≈5000 h	21	ISOS‐L‐2	P3HT: PCBM	3.54	≈2300	22
ISOS‐D‐2	P3HT: PC_70_BM	3.16	≈160 h	23	ISOS‐L‐1	PCDTBT: PC_70_BM	5.2	14 500	24
ISOS‐D‐2	DPPTTT: PC_70_BM	2.86	≈120 h	23	ISOS‐L‐1‐60% humidity	PTB7‐Th: PC_70_BM	10.5	>600	25
ISOS‐D‐1	PTB7: P(NDI2OD‐T2)	7.07	>72 days	26	ISOS‐L‐1	P3HT: PCBM	3.7	>6500	27

Another commonly used laboratory method to predict OPV lifetime is exposing devices to a constant irradiance, known as laboratory‐weathering tests. It is generally found that device lifetimes measured under dark storage are much longer than those measured when devices are irradiated. For some photosensitive organic semiconductors, e.g., PBDTTT‐EFT,^12^ device efficiency dropped to below 50% of its original value after several hours exposure to a solar simulator. This degradation is generally attributed to light‐induced photo‐chemical reactions occurring within the active layer of OPVs.

**Figure 1:**
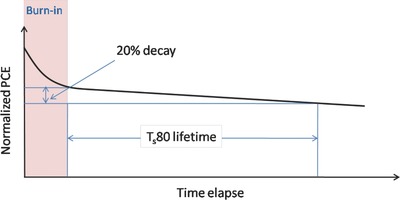
Typical degradation behavior of an organic solar cell.

A typical schematic of OPV efficiency as a function of time is shown in **Figure** **1**. Here, it can be seen that the device efficiency initially degrades rapidly under illumination.[[qv: 6b]] At a later point, this degradation rate slows and becomes more approximately linear. This initial, rapid degradation‐period is termed “burn‐in.”^13^ The lifetime of OPV devices are characterized by the lifetime parameter Ts80, which is extracted from the time point when the efficiency drops to 80% of its value at the end point of the burn‐in period. The end of the burn‐in process is defined as the end of the initial fast exponential decay or the start‐point of linear degradation.

Admittedly, in some cases the accurate determination of this point is not straightforward; however, in long‐term lifetime studies, the inaccuracy introduced by this uncertainty is relatively small. Sometimes the lifetime parameter T80 is quoted which is defined as the time over which the efficiency decays to 80% of its initial value. Clearly, Ts80 is longer than T80, as sometimes more than 20% of initial efficiency is lost during the burn‐in period. In many cases the T80 lifetime can be relatively short, however this does not necessarily result in a short Ts80.

The lifetime of an OPV module can be estimated by calculating the energy dose received by a module under indoor conditions. This is then converted to an equivalent energy dose that would be received from the sun under outdoor conditions. Peters et al.^14^ compared the stability of P3HT and PCDTBT based OPV devices held at their maximum power point exposed to a constant irradiance of 100 mW cm^−2^ (±4%) and a temperature of 37 °C (held using a water heated copper plate) over a period of 4400 h. For both types of device, a clear burn‐in period was observed lasting around 1300 h. Using a linear fit, a Ts80 lifetime of more than 12 000 h was extrapolated for PCDTBT based devices. It was also found that a clear determination of the end of the burn‐in period was critical in extrapolating the Ts80 lifetime. In theory, the end point of the burn‐in process should correspond to the turning point of the slope in the degradation curve after which efficiency degrades in a linear manner. However, identifying this point is subjective and a consensus should be established and applied to precisely define this end point. Indeed, by changing the end of the burn‐in process, the extrapolated Ts80 lifetime of P3HT based devices was varied from 5000 to 7000 h. Under the assumption that a PV device positioned outdoors would be exposed to an average irradiance level of one sun for 5.5 h day^−1^, a lifetime of 6.2 years and between 2.5 and 3.8 years was predicted for PCDTBT and P3HT based OPV devices respectively. Despite the relatively large errors that are associated with such extrapolations, a predicted lifetime of 6.2 years is an encouraging level of OPV stability. Furthermore, by minimizing oxygen and water exposure during the test conditions, Mateker et al.^15^ observed that OPVs could operate with minimal intrinsic degradation for thousands of hours, with extrapolated lifetimes extending beyond 15 years. The lifetime of several OPVs tested under the ISOS‐L standards is presented in Table 2. The references in Table 2 also show that optical‐radiation energy dose received by the OPV device is an important parameter in determining device lifetime.

**Figure 2:**
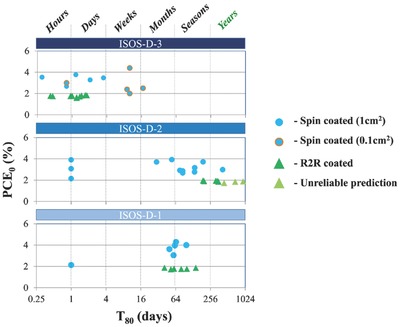
An “o‐diagram” displaying device lifetime obtained from different testing protocols. Reproduced with permission.^28^ Copyright 2014, Elsevier.

In some reports, device stability has not been estimated based on a single test, rather researchers have used a series of protocols to investigate the degradation of OPV devices. This raised the question of how to compare the lifetime data acquired under different protocols. Gevorgyan et al.^28^ established an “o‐diagram” method to present stability data in order to compare the lifetime determined under different testing methods and performed in different laboratories. This is shown in **Figure** **2**, where the *Y*‐axis of the o‐diagram represents the initial efficiency of an OPV module (either initial efficiency or efficiency just after the burn‐in process) and the *X*‐axis represents device lifetime plotted on a logarithmic scale. A second time‐scale presented at the top of the diagram divides time into hours, days, weeks etc. This presentation method is an effective way to compare device lifetimes obtained under different test protocols.

Recently, Kettle et al.^29^ established a lifetime testing model to obtain an acceleration factor for each of the ISOS standards that is defined as the ratio between device lifetime measured under accelerated and real world conditions. For acceleration factors less than 1.0, indoor‐tested devices degrade more slowly than those positioned outdoors. For factors greater than 1.0, indoor device degradation is accelerated compared to that determined under outdoor tests. In this study, it was concluded that the ISOS‐D‐1 testing condition resulted in an acceleration factor of 0.45. However with an increased temperature (ISOS‐D‐2) or an increased temperature and humidity (ISOS‐D‐3), the acceleration factor increased to 2.00 and 12.11 respectively. This suggested that elevated temperature and humidity significantly accelerates device degradation. Degradation under illumination was found to be generally faster than that determined under dark storage. Tests under the condition of ISOS‐L‐2 revealed an acceleration factor of 15.70. With the humidity elevated to 50%, the ISOS‐L‐3 condition resulted in an even larger acceleration factor of 24.70. Note such measurements were based on the outdoor conditions prevalent in Bangor, North Wales. The time required for different indoor lifetime testing protocols to simulate a one‐year outdoor degradation process is presented in **Table** **3**.

**Table 3:** Acceleration factors for ISOS‐D and ISOS‐L tests based upon the temperature–humidity model and temperature–light model, respectively. Included are the number of test hours required to simulate a 1‐year outdoor performance in Bangor, North Wales, using each ISOS test. Reproduced with permission.^29^ Copyright 2017, Elsevier

Testing standard	Temperature [K]	Relative humidity [%]	Irradiance [kW m^−2^]	Acceleration factor	Time required for 1 year outdoor simulation [h]
ISOS‐D‐1	298	50	0	0.45	19 393
ISOS‐D‐2	338	50	0	2.00	4377
ISOS‐D‐3	338	85	0	12.11	717
ISOS‐L‐2	338	n/a	1	15.70	558
ISOS‐L‐3	358	50	1	24.70	355

This work allowed lifetime data collected indoors under different ISOS standards to be related to expected lifetime under outdoor conditions. However, this model is clearly dependent on local climate conditions in North Wales and cannot provide a universal model to transfer indoor lifetime data to outdoor results. Indeed, due to the large variations in real‐world conditions, the establishment of a general model is not trivial. However, one possible solution is to determine a coefficient for each parameter; this will clearly require international coordination and collaboration together with considerable financial investment.

## 3 Outdoor Lifetime Tests

Considering the difficulties in simulating outdoor real‐world conditions for OPV lifetime tests, a number of researchers have explored moving such tests directly to outdoors. Indeed, outdoor lifetime tests are also included in the ISOS standard, as shown in **Table** **4**.

**Table 4:** Summary of ISOS standard for outdoor lifetime testing. Adapted with permission.^9^ Copyright 2011, Elsevier

Test type	Test ID	Light source (stress)	Temp.a)	R.H.b)	Environ.c)	Light source (test)
Outdoor	ISOS‐O‐1 Outdoor	Sunlight	Ambient	Ambient	Outdoor	Simulator
	ISOS‐O‐2 Outdoor	Sunlight	Ambient	Ambient	Outdoor	Sunlight
	ISOS‐O‐3 Outdoor	Sunlight	Ambient	Ambient	Outdoor	Simulator and sunlight

^a)^ Temperature

^b)^ Relative humidity

^c)^ Environment.

### 3.1 Test Platforms Used in OPV Outdoor Lifetime Study

**Figure 3:**
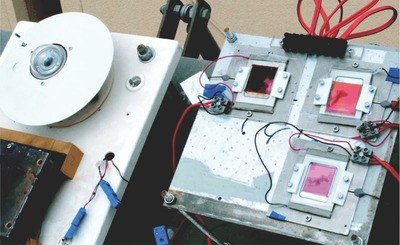
An early‐stage testing platform for OPV outdoor lifetime studies. Reproduced with permission.^30^ Copyright 2006, The European Physical Journal

To study OPV degradation outdoors, it is necessary to build a reliable testing platform. Such studies have been pioneered by F.C. Krebs and his colleagues, who have made strong progress in this area. In 2006,^30^ they reported the operational stability of OPVs based on three photovoltaic blends composed of the materials MEH‐PPV:PCBM, P3HT:PCBM, and P3CT:C60 in Israel (30.9°N). The equipment used was relatively simple, with a thermopile pyranometer and a thermocouple mounted with the OPVs under test in a solar tracker (see **Figure** **3**). The measurements were carried out in the daytime (from 9 a.m. to 5 p.m.), with devices stored in a nitrogen‐filled glovebox between tests. This periodic interruption meant the study was not comparable with subsequent outdoor lifetime studies, however the test protocol fulfilled other requirements of the ISOS‐O‐1 standard. Although the test only lasted for a month, it is still of great importance as it represents the first attempt to test OPV lifetime under real‐world conditions.

In 2008, researchers from Konarka Inc.^31^ established a more advanced outdoor lifetime testing platform in Lowell, USA (42.6°N) which was used to investigated the lifetime of flexible P3HT:PCBM OPV modules under outdoor conditions. The testing platform was located on a rooftop without any shade and faced south to maximize the solar irradiance. During the test, the OPV modules were kept under load conditions, and were connected to a resistor to make sure they operated at the initial maximum power point. The device outdoor lifetime performance was found to be promising, with no serious loss in performance determined after over 1 year's outdoor exposure. However, the maximum power point was found to shift and thereby induce a nonoptimal loading of the OPVs during testing. One important question raised by this study is the nature of the optimum load condition required for long‐term testing, and whether it is better to keep device under open circuit between the *J*–*V* measurements. Here, the setup fulfilled all requirements of ISOS‐O‐2 although it was reported prior to the establishment of the ISOS standards.

**Figure 4:**
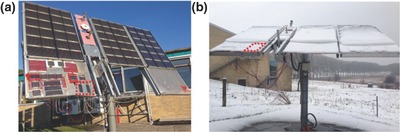
Outdoor lifetime testing platform in a) summer and b) winter. Reproduced with permission.^10^ Copyright 2015, Wiley‐VCH.

After the establishment of the ISOS standards, Krebs and co‐workers built a test platform located in Roskilde, Denmark (55.6°N). As shown in **Figure** **4**, the OPV modules tested were mounted on a solar tracker and connected with an automated system used to record a *J*–*V* curve every 10 min (and held at open circuit between measurements). Along with the device metrics, the system recorded environmental parameters including temperature and irradiance level. The OPV modules were intermittently dismounted from the platform and tested under a solar simulator to fulfill the requirement of ISOS‐O‐3. Their collaborators also built outdoor lifetime testing platforms in India, the Netherlands, Germany, Australia and Israel, which were simplified versions of the system in Denmark while still fulfilling ISOS‐O‐2 standards.

**Figure 5:**
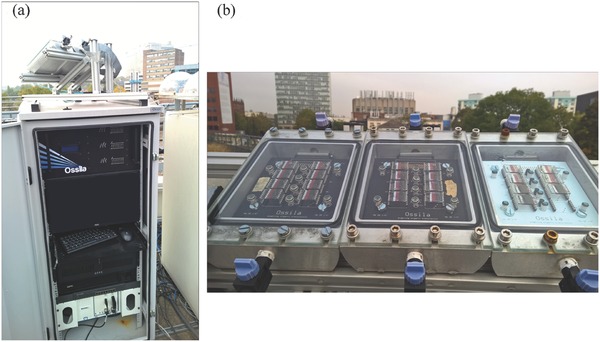
a) The rooftop lifetime testing system in Sheffield, UK. b) The sample chamber. Reproduced under the terms and conditions of the Creative Commons Attribution license 4.0.^32^ Copyright 2016, the authors.

Another outdoor lifetime testing platform was built in Sheffield, England (53.4°N).^32^ This system used a rigid sample chamber that provided an extra level of protection to the OPV modules (see **Figure** **5**). During operation, each sample chamber was filled with nitrogen at a slight overpressure to maintain devices in an inert atmosphere; a feature that made it possible to test OPV modules having relatively basic levels of encapsulation. The *J*–*V* curves were recorded at an interval of ≈5 min, with temperature and irradiance measured simultaneously. The sample chambers were held at an angle of 30° to the horizon and pointed south to maximize the solar flux incident upon the OPVs. Because of the use of the sample chamber however, this platform does not fulfill the requirement of ISOS‐O as the devices are no longer directly exposed to air or moisture. This is because the chamber does not form part of the device and cannot be considered as extra encapsulation. Nevertheless, it does allow long‐term comparison to be made between different organic‐semiconductor devices that have imperfect encapsulation.

**Figure 6:**
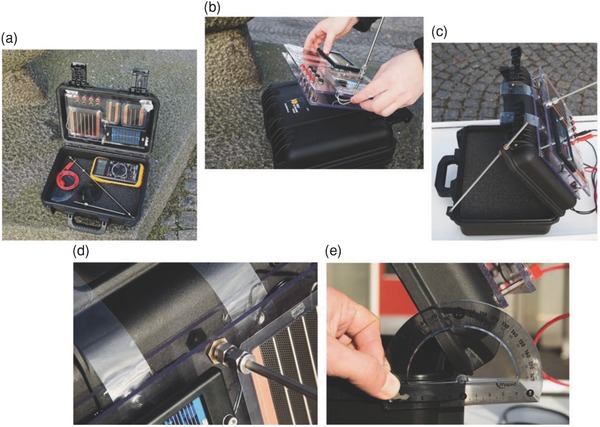
a) General view of the suitcase and its content; b) mounting of the sample platform on top of the suitcase; c) adjusting the angle of the lid via a rod with a thread; d) adjusting the angle to sun altitude; and e) measuring the angle. Reproduced with permission.^34^ Copyright 2014, Elsevier.

As the climate and geographical conditions significantly influence the performance and degradation of OPVs, it is useful to compare degradation of OPV modules located in different regions to explore the effect of climate on their long‐term outdoor stability. Krebs et al.^33^ conducted interlaboratory experiments by comparing outdoor lifetime data, however the systems used by different groups were not identical. Although the experiments were all designed to follow the ISOS‐O standard, small errors caused by the different setups cannot be ignored. To make outdoor lifetime studies easier and to increase the comparability of outdoor lifetime tests conducted by different groups, a standard testing platform is required. Krebs and co‐workers^34^ later designed a packaged outdoor OPV test suitcase, which served as both sample transportation and as a sample holder for outdoor testing. As shown in **Figure** **6**, the samples were mounted onto the outer surface of the suitcase, with the mini‐platform being fixed at a certain angle to optimize the absorption of the incident sunlight. The suitcase also provided the necessary electronics to determine open circuit voltage (*V*_oc_) and short circuit current (*J*_sc_). The development of this suitcase enabled comparable outdoor test experiments to be performed by most research laboratories and increased participation in the “OPV outdoor testing consortium”.

In summary, a number of successful long‐term outdoor lifetime testing platforms have been developed, however, a universal, cost‐efficient setup is still needed. The general requirements for such a platform include methods to automatically and continuously record *J*–*V* sweeps, temperature and irradiance level. Such systems are also portable and sufficiently inexpensive to be accessible to research groups having a limited budget. The establishment of such a test platform would require concerted action from the whole OPV research community.

### 3.2 Status of Long‐Term Outdoor Lifetime Testing

According to the database of ISI web of knowledge, a search including the key words “organic/polymer solar cells/photovoltaics,” returns more than 14 000 hits. However, when the key word “outdoor” is added to the search, only around 150 hits are found. Furthermore, the majority of OPV outdoor lifetime studies only last for a few hundred hours, with long‐term outdoor lifetime tracking studies being relatively rare.

Although short‐term outdoor lifetime testing cannot be used to extrapolate the long‐term lifetime of OPV modules, it is an effective tool to compare the influence of different designs on OPV stability. For instance, Teran‐Escobar et al.^35^ tested P3HT:PCBM based solar cells under outdoor conditions for a period of 1000 h in Barcelona, Spain. It was found the devices using a V_2_O_5_·0.5H_2_O HTL had good stability in outdoor conditions, with the use of a UV filter being beneficial in improving device stability (UV irradiance can induce photoreactions and thereby reduce device performance). A similar study was conducted by the same group,^36^ where an outdoor lifetime study was conducted for 900 h following the ISOS‐O‐2 standard. Here, it was found that the use of an aqueous solution‐processed V_2_O_5_ hole transport layer could improve P3HT:PCBM based OPV module lifetime, with devices still retaining more than 80% of their initial efficiency after 900 h of continuous testing. Josey et al.^37^ tested the outdoor stability of some fullerene‐free OPV devices over around 40 days and concluded that the chemical structure of the acceptor molecule had significant impact on device stability. Due to the restriction of the testing platform used, the samples were only exposed to outdoor conditions for 6 h day^−1^ and were returned to indoor conditions for dark storage at night, and thus this study cannot be directly compared with other work.

Most outdoor studies have been conducted by Krebs and co‐workers, with a particular focus on P3HT:PCBM based solar modules fabricated by roll‐to‐roll processing methods. Their outdoor lifetime studies have been performed in different counties including Denmark, India, Holland, Germany, Israel, and Australia. The details of their results are presented in **Table** **5**. Other groups have also reported long‐term outdoor lifetime studies of OPV devices in different locations. For example, Emmott et al.^38^ studied the off‐grid stability of OPV modules in outdoor conditions in Rwanda, Africa. The outdoor stability in Africa—where the UV levels and ambient temperature are much higher than Europe—was determined to be between 2.5 and 5 months; a value smaller than that of the same module tested in Europe. The failure of the encapsulation was identified as the main cause of the degradation. Krebs and co‐workers have also explored OPV module lifetime in a greenhouse^39^ and found that module lifetime was enhanced slightly; a result that suggests possible new applications for OPVs.

**Table 5:** Summary of reported long‐term outdoor lifetime testing of OPVs

Test protocols	Location	Material	Initial PCE [%]	Test duration	Lifetime	Ref.
ISOS‐O‐2	Lowell, USA	P3HT:PCBM	>1	20/09/2006–07/11/2007	>1 year	30
ISOS‐O‐3	Roskilde, Denmark	P3HT:PCBM	≈2	10/10/2012–10/10/2014	>2 year	10
ISOS‐O‐2	Roskilde, Denmark	P3HT:PCBM	>1.5	03/10/2012–09/10/2013	>1 year	[[qv: 33a]]
ISOS‐O‐2	Roskilde, Denmark	P3HT:PCBM	0.72–2.24	17/5/2013–03/12/2014	Weeks to seasons	43
ISOS‐O‐3	Germany, Israel, Australia, Denmark	P3HT:PCBM	0.7–1.4	06/2011–11/2012	Hundreds to 10 000 h	[[qv: 33b]]
ISOS‐O‐2	Denmark	PBDTTTz‐4:PCBM	1.01–1.9	11/07/2014–	>1000 h	44
ISOS‐O‐1	Denmark	P3HT:PCBM	≈1.5	05/2014–09/2014	≈1000 h	45
ISOS‐O‐2	Victoria, Australia	P3HT:PCBM	≈0.85	02/2014–03/2015	>1 year	42
ISOS‐O‐2	Southern Rwanda	P3HT:PCBM	0.35	1/09/2014– 30/05/2015	2.5–5 months	38
ISOS‐O‐3	Sheffield, England	PCDTBT:PC_70_BM	5.04–6.24	18/09/2014– 20/09/2015	5200–6200 h	32
ISOS‐O‐3	Sheffield, England	PFD2TBT8:PC_70_BM	5.9	03/12/2014–03/04/2016	>10 000 h	46

The lifetime of OPV devices is significantly affected by the quality of the encapsulation;^40^ this is especially true in outdoor applications as devices are exposed to a range of stresses including irradiance, thermal cycles, wind, rain, snow, and high moisture‐levels.^10^ It has been shown that unencapsulated devices have operational lifetimes that are several magnitudes lower than encapsulated ones.[[qv: 5c]] Although the importance of encapsulation has been well established, the packaging of OPV modules is around 60% part of their total cost.^41^ The development of secure, inexpensive and effective encapsulation packages remains a real challenge. Weerasinghe et al.^42^ developed an encapsulation strategy based on commercial available barrier films and adhesives and used this to package fully printed OPV modules that showed limited efficiency loss after 13 months outdoor operational testing. The modules experienced harsh weather conditions during outdoors testing, including ambient temperatures ranging from −1 to 45 °C, heavy rain and hailstorms. Control, nonencapsulated modules were found to be completely nonfunctioning within 48 h of outdoor exposure even without being exposed to any “extreme” weather. The study clearly shows that the intrinsic stability of all‐printed OPV modules is highly promising and provides significant motivation to develop more effective and cheap encapsulation techniques that can be used to protect large‐area and flexible OPV modules.

As can be seen from Table 5, OPVs tested outdoors have demonstrated lifetimes exceeding 2 years provided they are effectively encapsulated. However, outdoor lifetime tests conducted over longer times periods are still required. Most reported long‐term OPV outdoor lifetime tests are based on devices containing an active layer composed of P3HT:PCBM, a material system that is known to have high intrinsic stability. Progress has been made in the development of flexible OPV modules having promising stability when tested under outdoor conditions.^10^ Here, the concept of the water vapor transmission rate (WVTR) is of key importance. This parameter is used to characterize the amount of water vapor that passes through a layered material over a set time period and has units of g m^−^² day^−1^.^47^ We note that it has been proved challenging to develop long‐lived flexible organic LEDs for display applications.^47^ This suggests that a less demanding WVTR is required for OPV applications as compared to OLEDs (see discussion in Section 4.3).

It has been argued that low OPV module efficiency is not an obstacle for commercialization providing that devices cover a sufficiently large area and that manufacture cost is sufficiently low.^48^ However, high power conversion efficiency is always desirable as this will reduce the energy payback time. OPV modules have been fabricated using D–A polymer:fullerene systems having much higher PCE.^49^ Indeed, the authors of this review have used two such materials and have performed outdoor lifetime studies, with device lifetimes demonstrated between 6200 and 10 000 h.^47^, ^61^ More efficient donor materials and nonfullerene acceptor materials have advanced the PCE of OPV devices to more than 10%, however, most of the stability research on these materials is still limited to laboratory conditions.^50^ More work is needed to move the stability testing to outdoor conditions.

The adoption of ISOS‐O standards clearly results in compatibility between tests conducted by different research groups. Although such ISOS‐O standards are detailed, Gevorgyan et al.[[qv: 33b]] made a series of further suggestions and supplements to such measurements that we summarize here:

(1) To ensure the reproducibility and reliability of the lifetime data, at least 5 identical devices should be measured under the same conditions.
(2) The environmental conditions including temperature, humidity, and irradiance level should be monitored and recorded along with OPV device metrics.
(3) The cumulative energy dose received by the samples should be calculated over the whole test period.
(4) Samples should be periodically taken back to laboratories and tested under well‐defined indoor conditions (at least once a month is recommended). This is especially necessary in winter or in rainy seasons when irradiation is limited. However, mechanical and electrical stresses during such indoor tests should be carefully controlled and minimized.
(5) As the irradiance level has great influence on the device efficiency, the data collected should be screened according to specific irradiance level range. The *J*_sc_ should be normalized to the irradiance level to make a fair comparison.
(6) If possible, the efficiency and temperature coefficient of the device should be established and the PCE should be corrected according to this coefficient.
(7) A direct link between the ISOS‐L and ISOS‐O lifetime tests should be established via the cumulative energy dose received by the devices,^46^ allowing a comparison to be made between indoor and outdoor lifetime data. Another effective way to compare indoor and outdoor lifetime data is through “o‐diagram” as described by Gevorgyan et al.^43^

## 4 Outdoor Factors Influencing OPV Device Stability

The environment is a dynamic system, with temperature, humidity and irradiance levels all changing simultaneously over time and over seasons. In the following sections, we discuss how these factors influence OPV lifetime.

### 4.1 Temperature

The efficiency of OPVs is strongly dependent on temperature, as charge transport in organic semiconductors occurs through a thermally‐assisted hopping process^51^ and thus short circuit current (*J*_sc_) usually increases with elevated temperature. The open circuit voltage (*V*_oc_) decreases slightly with increased temperature,^52^ which can be expressed using the following equation

(1) $$V_{\mathrm{oc}}=\frac{1}{e}\left(E_{\mathrm{LUMO}}^{A}-E_{\mathrm{HOMO}}^{D}-\Delta \right)-\frac{kT}{e}\ln\left(\frac{n_{e}n_{h}}{N_{c}^{2}}\right)$$

**Figure 7:**
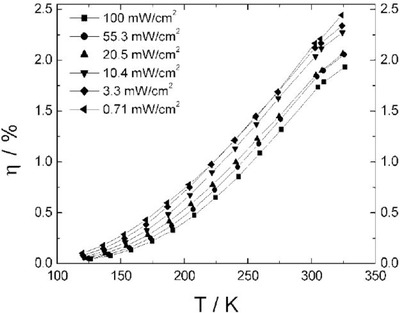
Device PCE as a function of temperature under different irradiance levels. Reproduced with permission.^53^ Copyright 2004, Wiley‐VCH.

Here, Δ is related to disorder resulting from the solution processed and phase separated polymer and fullerene regions, *n*_e_ and *n*_h_ are the electron and hole densities in the acceptor and donor domains at open circuit, and *N*_c_ is the density of conduction states (DOS) at the band edge of the acceptor and donor. The overall device efficiency most often increases due to a stronger positive correlation of *J*_sc_ with temperature. It has been shown that the efficiency of ITO/PEDOT:PSS/OC_1_C_10_‐PPV:PCBM/Al OPV devices increases from below 0.8% at 250 K to 1.9% at 320 K as shown in **Figure** **7**.^53^ The same phenomenon has been reported in OPVs employing MDMO‐PPV:PCBM as the photoactive layer.^54^ However recent studies based on tracking the diurnal performance of small‐molecule planar‐mixed heterojunction DBP:C_70_ OPV devices in outdoor conditions suggested that the positive temperature coefficient resulted from spectral broadening of the absorption caused by enhanced electron–phonon coupling at elevated temperatures which increased *J_sc_*.^55^

**Figure 8:**
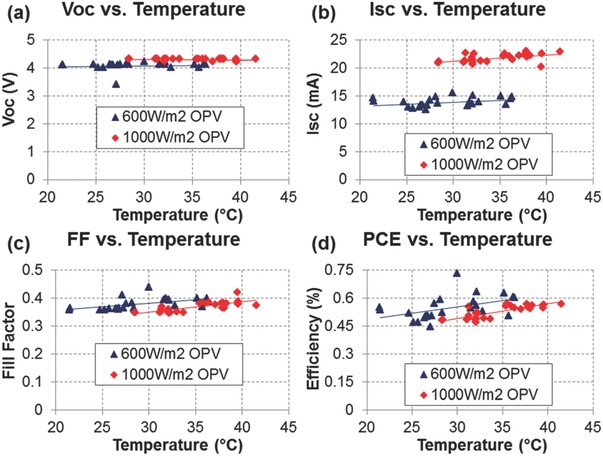
Temperature dependence of a) *V*_OC_, b) *J*_SC_, c) Fill Factor, and d) PCE measured at fixed irradiances of (600 ± 10) W m^−2^ and (1000 ± 10) W m^−2^. Fits show linear curves that characterize the temperature coefficient of the module. Reproduced with permission.^56^ Copyright 2015, American Institute of Physics.

Practically, it is important to understand the effect of temperature up to around 60 °C as this covers the temperature range encountered in most real‐world situations. Over the course of a single day, variations in temperature can significantly affect device efficiency and thus a temperature coefficient can be determined to minimize efficiency fluctuations induced by changing temperature.^56^ As can be seen in **Figure** **8**, device efficiency has a positive coefficient with temperature when measured under outdoor conditions. However, such temperature coefficients are largely dependent on the composition of the active layer and the device architecture, and such a temperature coefficient must be independently established for each type of device. Unfortunately, device efficiency is not routinely corrected for the effect of temperature in most reported outdoor lifetime studies.

The ambient temperature also affects OPV lifetime. As described previously, OPV device degradation is accelerated by elevated temperature; a process reflected by Equation (2), 57

(2) $$AF=\left(\frac{I_{1}}{I_{2}}\right)\exp\left[\frac{E_{a}}{k_{B}}\left(\frac{1}{T_{2}}-\frac{1}{T_{1}}\right)\right]$$

Here *AF* is the acceleration factor that occurs as a result of increased temperature and irradiance level, *E*_a_ is the activation energy of the degradation process and *k*_B_ is Boltzmann constant with *T*_1_ (*I*_1_) and *T*_2_ (*I*_2_) being temperature (irradiance level) under testing conditions (1) and (2) respectively. This simplistic model makes the following assumptions: 1) the activation energy *E_a_* value over the temperature range is constant, 2) the rate of degradation depends linearly on irradiance, and 3) the spectral composition (especially UV content) of the radiation is unchanged at different irradiance levels.^58^ Aging tests on P3HT:PCBM solar cells have confirmed the validity of this relationship and have established an acceleration factor of 4.45 over a storage temperature range from 298 to 333K.^59^ However, under outdoor conditions with the presence of irradiance, photo‐oxidation is the dominant degradation mechanism rather than thermally induced oxidation, and thus the influence of temperature will mainly occur via its effect on the rate of photochemical reaction.^60^

In recent years, the emergence of nonfullerene acceptor materials has increased the PCE of bulk heterojunction OPV devices.[[qv: 3h,61]] Besides the high efficiency, another advantage of fullerene‐free OPV devices is excellent thermal stability. OPV devices using an unfused‐core based nonfullerene acceptor, DF‐PCIC, realized a PCE of 10.2%, and more importantly, after thermal treatment at 180 °C for over 12 h the devices retained ≈70% of their original efficiency.^62^ Similarly, OPV devices based on ITIC, another nonfullerene acceptor small molecule also showed excellent thermal stability.^63^ Under thermal stress of 100 °C for 100 h, no obvious efficiency loss was observed. Due to the strong tendency of fullerene derivatives to form large aggregates at high temperatures,[[qv: 58b]] OPV devices using fullerene acceptors generally have poor thermal stability. Replacing the fullerene acceptor by nonfullerene acceptors can avoid the morphological instability caused by fullerene aggregation at high temperature and so result in improved thermal stability. We note that in outdoor conditions (especially in some tropical regions), high stability at elevated temperature is essential. Replacing fullerene acceptors by nonfullerene molecule is therefore a promising strategy to extend device lifetime, although a detailed investigation of the stability of such materials to other degradation mechanisms is still needed.

### 4.2 Irradiance Level

**Figure 9:**
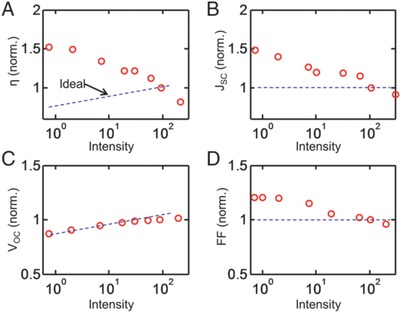
Irradiance‐dependent performance of an OPV device as a function of irradiance level. All performance metrics are normalized to values determined at an intensity of 100 W cm^−2^. Dotted lines correspond to results from the self‐consistent numerical simulations for typical inorganic solar cells. Reproduced with permission.^64^ Copyright 2015, National Academy of Sciences of the United States of America.

The irradiance level both affects device metrics and also accelerates the device degradation rate. Ideally, the normalized *J*_sc_ and fill factor (FF) should be constant as a function of irradiance level as charge generation is proportional to the light intensity. Under open circuit condition, all photogenerated charge carriers recombine within the device. Thus, the recombination mechanisms can largely determine *V*_oc_ of OPVs. As shown in **Figure** **9**c,^64^ *V*_oc_ varies logarithmically with illumination intensity, with its slope being equal to *kT*/*e*. From Equation (1), it can be seen that *V*_oc_ is particularly susceptible to the density of states (DOS) of the acceptor LUMO and donor HOMO. The DOS in the band tails is dependent on the illumination intensity as such states can be occupied by photoexcited electrons (in the acceptor) and holes (in the donor). At temperatures above zero, the quasi‐Fermi energies move into the gap thereby reducing the *V*_oc_. Based on the above discussion, the overall device efficiency will increase with increasing irradiance intensity; a process that is observed in silicon‐based solar cells. In an OPV however, charge carriers are generated through the processes of photon absorption, exciton diffusion, and separation followed by charge extraction. A higher irradiance level normally results in a higher exciton generation rate, although not all generated excitons undergo separation, as some fraction are lost through monomolecular or bimolecular recombination.^65^ The short circuit current is linearly proportional to the irradiance level, however carrier‐traps in the active layer significantly influence the dependence of *J*_sc_ on the irradiance level. At a high light intensity, more traps become populated, resulting in reduced recombination and superlinear increase of the photocurrent.^66^ The open circuit voltage is expected to be proportional to the light intensity over the temperature range 280 to 320 K,^54^ a temperature that coincides with most outdoor conditions. It is also found that the parallel resistance of OPVs decreases by almost three orders of magnitude as the irradiance level is increased from 0.03 to 100 mW cm^−2^. However the overall device efficiency decreases slightly with increased irradiance level due to the negative effect of decreased parallel resistance. Similar results were observed on OPV devices based on a squaraine dye,^67^ with PCE increasing from 4.3% at 100 mW cm^−2^ to 6.2% at 3.5 mW cm^−2^ because of increased FF. It was believed that at a lower irradiance level, recombination was suppressed due to a lower charge carrier density in the device.

A collection‐limited theory also confirmed the dependence of device efficiency on irradiance level, as shown in Figure 9.^64^ Here, it was found that the space‐charge density increased with increasing irradiance level. This increase in space charge with increasing illumination intensity pointed to a filling of deep‐level charge‐traps present in the material. These filled deep‐level traps can screen the electric field and thus reduce the charge extraction efficiency.

It is worth noting that under outdoor conditions, higher irradiance levels usually correspond to higher temperatures, an issue that makes it difficult to distinguish between codependent factors. The effect of irradiance on device performance under outdoor conditions was investigated by Bristow et al.^56^ Here, it was found that at low irradiance, device efficiency was much lower than expected and only reached a maximum at 600 mW cm^−2^, with a clear inflexion characteristic observed in the *J*–*V* curve. It was speculated that there was poor carrier transport through one of the layers or interfaces that prevented efficient charge‐extraction from the device. This study clearly illustrates the complexity of outdoor testing of OPV devices, with unexpected results sometimes emerging due to the combined effects of a number of environmental factors.

**Figure 10:**
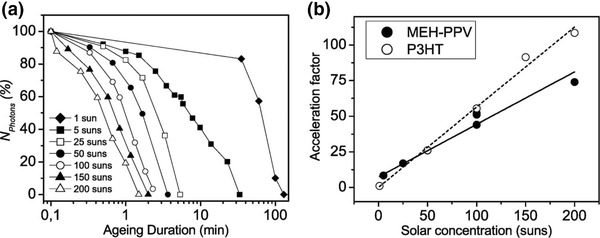
a) Degradation of MEH‐PPV expressed as a decrease of the total absorption. b) Acceleration factors for MEH‐PPV and P3HT at different solar intensities. Reproduced with permission.^68^ Copyright 2011, Elsevier.

Data collection times in OPV lifetime tests can be shortened by exposing devices to concentrated illumination. In order to investigate the intrinsic degradation mechanisms of organic semiconductor materials (rather than complete devices), Tromholt et al.^68^ studied the degradation of P3HT and MEH‐PPV at varied irradiance levels (between 20 and 100 W cm^−2^). Here the total absorption was recorded using UV–visible spectroscopy as a function of exposure time at different illumination levels. As shown in **Figure** **10**, it was found that when exposed to concentrated illumination, the degradation of both polymers was accelerated, with the acceleration factor being almost linear with irradiance level.

Although the active layer is the most sensitive part of an OPV device, the degradation of electron and hole transport layers, the device‐electrodes and interfaces also need to be considered. For example, Tromholt et al.^69^ investigated the degradation of OPV devices based on a P3HT:PCBM blend as active layer and found that device efficiency dropped to 6% of its original value after exposing the device at a constant irradiance of 500 mW cm^−2^ for 30 min. This degradation was attributed to the desorption of oxygen from the zinc oxide electron transport layer during illumination. The study indicates therefore that sensitivity to other materials within the device is critical to engineer enhanced operational stability, and that performance at high irradiance level can reveal degradation mechanisms that are not observed under normal irradiance conditions. Indeed, under outdoor conditions, the irradiance level seldom reaches values as high as 150 mW cm^−2^, with the average irradiance level being much less than 1 sun. Degradation mechanisms that only occur at high irradiance level are therefore of secondary importance in outdoor lifetime tests.

### 4.3 Humidity

Moisture is a key degradation factor for OPVs. Glen et al.^70^ found that moisture plays an important role in the degradation of OPV devices incorporating PEDOT:PSS/ITO and Ca/Al electrodes, with devices exposed to humid air degrading more rapidly than those exposed to dry air. Water was shown to cause the formation of bubbles and voids within the device. It was also concluded that water ingress mainly occurred via the edge of the device rather than through pinholes or defects in the aluminum electrode. This finding emphasized the need for effective encapsulation at the edges of an OPV module.

**Figure 11:**
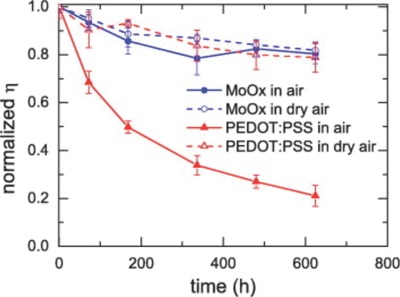
Normalized efficiency degradation of devices with either PEDOT:PSS (red triangles) or MoOx (blue circles) as a hole transport layer for devices stored under ambient conditions (≈35% RH) and dry air (<5% RH). Reproduced with permission.^71^ Copyright 2011, Elsevier.

Devices incorporating a PEDOT:PSS layer are believed to be more vulnerable to the effects of moisture because of its hygroscopic nature. Voroshazi et al.^71^ investigated the degradation of P3HT:PCBM based OPV devices incorporating either PEDOT:PSS or MoO_3_ hole transport layer, with the results revealing that moisture induces significant degradation in devices containing a PEDOT:PSS layer. Devices that incorporated a MoO_3_ hole transport layer however appeared relatively stable even in atmosphere containing moisture (see **Figure** **11**). Similar results were reported by Sun et al.^72^ who explored PCDTBT:PC_70_BM based OPV devices and found that by replacing the PEDOT:PSS hole transport layer with MoO_x_, it was possible to significantly increase the device air storage stability. Here, devices incorporating a MoO*_x_* hole transport layer retained 50% of their original efficiency after 720 h air storage without encapsulation. The efficiency of control devices incorporating a PEDOT:PSS hole transport layer instead degraded more rapidly, retaining less than 10% of their original value after air storage for 480 h. However for encapsulated PCDTBT:PC_70_BM based OPV devices, Bovill et al.^24^ reported that PEDOT:PSS hole transport layers resulted in improved device stability under long‐term illumination testing in air compared to devices using MoO*_x_* or V_2_O_5_ hole transport layers. It is possible that the difference between these findings result directly from differences in test conditions; studies conducted under full illumination condition (rather than dark storage) generally result in higher ambient temperatures which help to remove residual moisture from the PEDOT:PSS and the surrounding device by evaporation. In such circumstances, the hydroscopic nature of the PEDOT:PSS hole transport layer may be of secondary importance. Further work is needed to clarify such issues.

Avoiding the ingress of moisture is essential to create stable OPV modules. It has been shown that the WVTR should be less than 10^−6^ g m^−2^ d^−1^ in OLEDs to achieve suitable lifetimes.^73^ However, the global standard for OPV devices has not yet been established. For OPV devices having relatively stable electrodes, Cros et al.^74^ showed that a WVTR of 10^−3^ g m^−2^ d^−1^ was necessary to obtain a lifetime of several years. This less demanding WVTR requirement for OPVs points favorably to the use of low cost encapsulation solutions.

Interestingly, replacing fullerene acceptors by nonfullerene acceptor molecules can also increase the air storage stability. Using a nonfullerene acceptor, O‐IDTBR, P3HT based solar cells exhibited an efficiency of 6.4%, which is even higher than fullerene based P3HT solar cells. More importantly, the stability under ambient dark storage condition of O‐IDTBR:P3HT devices was determined to be superior to other fullerene based OPV devices.[[qv: 50a]] The first 60 h witnessed a fast degradation and then PCE remained relatively stable and retained 73% of the initial PCE after 1200 h ambient dark storage. This result confirmed the good stability of fullerene free OPV devices against water and oxygen in the ambient atmosphere.

### 4.4 Thermal Fluctuations

**Figure 12:**
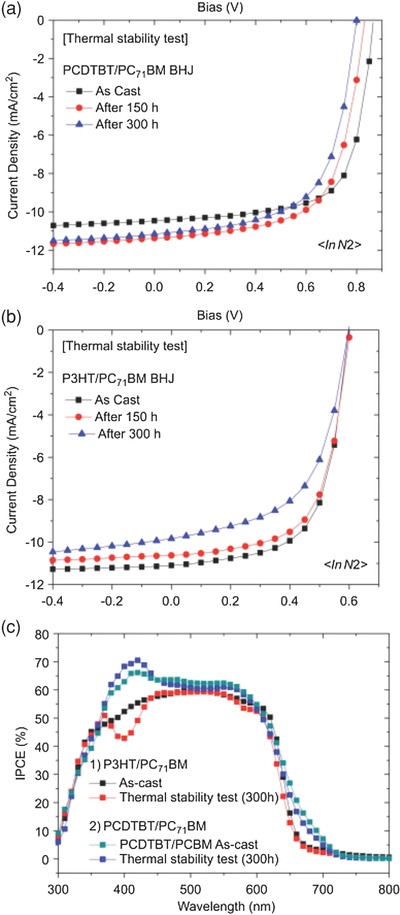
*J*–*V* curves of BHJ devices with a) PCDTBT/PC_71_BM and b) P3HT/PC_71_BM as a function of storage time (300 h) following a thermal stability test in N_2_ and c) IPCE spectra of the devices with P3HT/PC_71_BM or PCDTBT/PC_71_BM before and after thermal stability tests. Reproduced with permission.^76^ Copyright 2011, Elsevier.

Thermal fluctuations are a natural consequence of outdoor lifetime testing, with this process also contributing to the degradation of OPV devices. For this reason, thermal cycling tests form an essential component of tests applied to commercially available PV.^75^ In outdoor conditions, ambient temperatures can vary over 20 °C in a single day, with such fluctuations being even larger in certain geographic locations. To explore the importance of thermal fluctuations on OPV stability, Wang et al.^76^ alternated the storage temperature of PCDTBT‐ and P3HT‐based OPVs between 80 and 25 °C every 12 h over a total period of 300 h. It was found that PCDTBT and P3HT based devices retained 90% and 80% of their original efficiency respectively (see **Figure** **12**). This test was conducted under a nitrogen atmosphere in the dark. It is believed^77^ that under outdoor conditions, the degradation caused as a result of thermal fluctuations will be enhanced by the presence of oxygen, moisture and illumination. Indeed, the effect of thermal cycling on the device efficiency and mechanical integrity of P3HT:PCBM based OPV devices has been investigated under even harsher conditions.^78^ Here, it was found that thermal cycling between −40 and +85 °C at a heating/cooling rate of ≈1.4 °C min^−1^ over 200 cycles caused device efficiency to decrease from ≈2.0% to ≈1.5% after the first 5 cycles, with efficiency remaining constant afterward.

## 5 Burn‐In Process in OPV

Figure 1 plots a typical degradation curve of an OPV device. Here, the efficiency undergoes an initial, rapid period of degradation that is termed as “burn‐in.” The efficiency loss during burn‐in varies for different materials; for example an efficiency loss of up to 40% was observed in PCDTBT based OPV devices during burn‐in,^14^, ^15^, ^20^ while this is as much as 60% in PBDTTT‐EFT based OPV devices.^12^

The OPV burn‐in process is related to device irradiation, as no obvious burn‐in is observed under dark storage.^13^ Origins of burn‐in loss have been attributed to photo‐induced reactions in the active layer and the formation of sub‐band gap states.^13^ Such sub‐band gap states in OPV devices are believed to reduce *J*_sc_ and *V*_oc_ in two ways. First, they increase the recombination rate, reduce the exciton lifetime and diffusion length and thus reduce steady state charge carrier density.^79^ The charge carrier density is directly related to *J*_sc_. Secondly, charge carriers can fill sub‐band states near the quasi‐Fermi level. Even though this does not change the total charge carrier density,^80^ such sub‐band gap states can still result in *V*_oc_ loss.^81^ This is reflected in Equation (1), as the quasi‐Fermi levels move away from donor HOMO and acceptor LUMO levels and into the energy gap between donor HOMO and below acceptor LUMO levels.^82^

**Figure 13:**
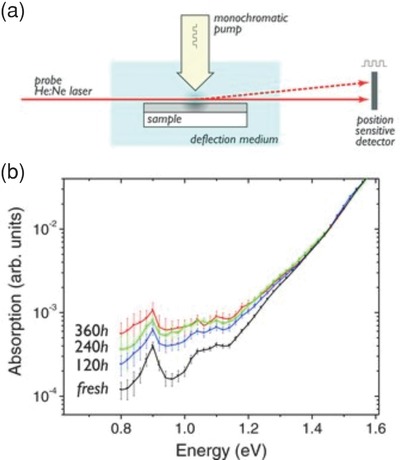
Photothermal deflection spectroscopy (PDS) of PCDTBT:PC_70_BM films. a) Schematic of the PDS set‐up. b) PDS absorption spectra of fresh and aged films, where the absorption below 1.3 eV increases during aging at a similar rate to the decrease in solar cell efficiency during burn‐in. Reproduced with permission.^13^ Copyright 2012, Wiley‐VCH.

The formation of sub‐band gap states has been confirmed using photothermal deflection spectroscopy (PDS).^13^ Here, PCDTBT:PC_71_BM blend films were deposited on a quartz substrate and exposed to 1 sun equivalent irradiance. PDS absorption spectra were then periodically measured and compared with an unexposed control film. As shown in **Figure** **13**, an increased absorption was observed in the energy region below 1.2 eV and indicated the formation of sub‐band gap states. As can be seen, this absorption increase occurs most rapidly during the first 120 h exposure and changes at a similar rate to the decrease in solar cell efficiency observed during burn‐in. During the next 240 h, the increasing rate slowed down with the device efficiency also degrading at a slower rate. This indicates that the “burn‐in” process lasts for around 120 h and has the same origin as the absorption enhancement below 1.2 eV in the PDS spectra.

Photo‐induced dimerization of fullerenes is another possible origin of device burn‐in, as this reduces the active‐layer exciton‐harvesting efficiency and thus results in a loss in the short circuit current density. It has been shown that the external quantum efficiency (EQE) loss after exposure to illumination mainly corresponds to the reduced absorption of the fullerene.^83^ In a dimerized fullerene, excitons are trapped in the fullerene phase and cannot be separated and collected efficiently; a process resulting in a reduced *J*_sc_. By replacing PCBM with the nonfullerene acceptor rhodanine‐benzothiadiazole‐coupled indacenodithiophene (IDTBR),^84^ P3HT:IDTBR based OPV devices lost only 5% of relative PCE after exposure to 1‐sun equivalent irradiance over the course of 2000 h. This degradation rate is significantly less than that of P3HT:PCBM devices, which under the same test conditions underwent a relative PCE loss of 34% PCE. This indicates that the use of nonfullerene acceptors may be an effective strategy to increase the stability of OPV devices.

In PffBT4T‐2OD:PCBM based OPV devices,[[qv: 51b]] an abnormally strong burn‐in degradation has been observed, with the PCE dropping from 9.20% to 5.62% after dark storage for 5 days. Here, demixing of the donor/acceptor mixed‐phase within the BHJ film was attributed to be the cause of this considerable efficiency loss. Such spontaneous phase separation in mixed amorphous regimes can occur at room temperature and is independent of storage conditions. The authors claimed that this phenomenon is highly dependent on the material combination used in the BHJ film. This study indicates that not all OPV burn‐in losses are photo‐induced; rather morphological evolution is also a potential degradation mechanism in some specific material systems. In contrast, Pearson et al.^12^ working on PBDTTT‐EFT:PC_71_BM based OPV devices observed that the nanostructure of the active layer and kinetics of free charge generation were apparently unchanged after burn‐in, and thus the initial degradation of device efficiency was attributed to generation of charge trapping states and suppressed charge carrier dissociation. Clearly, the morphological evolution of each BHJ system is highly dependent on the molecular structure of the particular materials used, with more work required to bring the different observations into a coherent framework.

**Figure 14:**
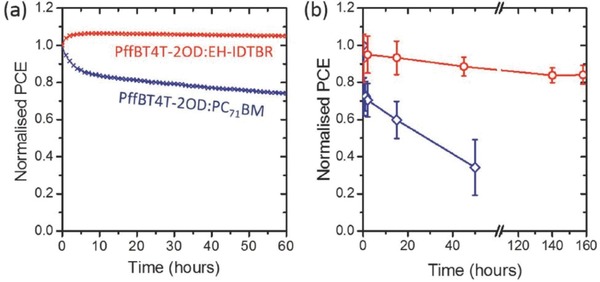
Normalized PCE of PffBT4T‐2OD:EH‐IDTBR devices during a) light soaking without UV light, with devices maintained at a temperature below 50 °C, and b) during annealing at 85 °C in a nitrogen atmosphere. Reproduced with permission.^85^ Copyright 2017, Wiley‐VCH.

Interestingly burn‐in losses are nearly negligible if the fullerene acceptor in PffBT4T‐2OD based OPV devices is replaced with a nonfullerene derivative.^85^ For example, PffBT4T‐2OD:EH‐IDTBR based OPV devices showed no degradation under constant irradiance stress for over 60 h, with devices having promising stability under a thermal stress of 85 °C (See **Figure** **14**); a result pointing to a promising morphological stability of nonfullerene based PffBT4T‐2OD based OPV devices. The improved stability against photo‐induced burn‐in loss of PffBT4T‐2OD:EH‐IDTBR OPV devices is attributed to greater resistance to photo‐induced electronic trap state formation compared to devices incorporating a PC_71_BM acceptor. These results suggest better stability of fullerene free OPV devices over those using fullerene acceptors. However, the light soaking experiments lasted for only 60 h, which makes it impossible to extract the Ts80 lifetime of the fullerene free OPV devices and so a direct comparison of the published data cannot be made.

## 6 Summary and Outlook

We have reviewed the status of outdoor lifetime studies of OPVs. The reported outdoor operational lifetime of certain OPV modules has now reached a period of several years; a promising result considering that 10 years ago, typical device lifetimes were in the range of a few days to weeks.

OPV lifetime studies conducted under laboratory conditions were briefly reviewed. The “o‐diagram” methodology and accumulated energy dose analysis can be used to make comparisons between indoor and outdoor lifetime studies, however indoor‐based tests do not fully simulate the outdoor environment. Direct measurements of OPV outdoor lifetime were reviewed. Here we discussed the development of experimental systems used in outdoor lifetime studies, with recommendations made to increase the consistency of different outdoor lifetime tests. Long‐term outdoor lifetime test results for different OPV material‐systems were then summarized. It was highlighted that certain OPV modules fabricated using roll‐to‐roll processes and encapsulated using flexible PET foils have very promising operational stability when measured under outdoor conditions. In the majority of studies however, OPVs are fabricated using nonscalable techniques and have a limited active area. Nevertheless, such studies are useful in exploring the intrinsic stability of OPV materials and devices when exposed to different geographic locations and climatic conditions. In outdoor lifetime conditions, the irradiance level, temperature, humidity, and thermal fluctuation have been identified as key degradation factors, with their influence on OPV performance and stability discussed. Finally, the burn‐in phenomena observed during the initial period of OPV operation is introduced, with burn‐in free OPVs based on nonfullerene acceptors being highlighted. The stability of fullerene free OPV devices looks promising based on current research results, especially under thermal stress and light soaking. However, more systematic investigation is needed and outdoor lifetime studies of devices with nonfullerene acceptors are needed.

Although considerable progress has been made in outdoor lifetime testing of OPVs, there are still some challenges that remain including the development of a standard outdoor lifetime testing platform and testing strategy. In addition, a comprehensive, predictive method to fully link lifetime tests conducted under indoor (accelerated) conditions to outdoor real‐world conditions should be developed. In general, outdoor lifetime testing is generally limited to the most well‐established material systems (such as P3HT:PCBM and PCDTBT:PC_70_BM), and thus it will be interesting to extend it to new donor/acceptor blends having high efficiency—even if such tests are initially performed over a limited period under the ISOS‐O‐1 basic testing protocol.

## Conflict of Interest

The authors declare no conflict of interest.
